# Serum bilirubin levels are associated with poor functional outcomes in patients with acute ischemic stroke or transient ischemic attack

**DOI:** 10.1186/s12883-021-02398-z

**Published:** 2021-09-27

**Authors:** Quping Ouyang, Anxin Wang, Xue Tian, Yingting Zuo, Zhimeng Liu, Qin Xu, Xia Meng, Pan Chen, Hao Li, Yongjun Wang

**Affiliations:** 1 China National Clinical Research Center for Neurological Diseases, Advanced Innovation Center for Human Brain Protection, Beijing Tiantan Hospital, Capital Medical University, No.119 South 4th Ring West Road, Fengtai District, 100070 Beijing, China; 2 Department of Neurology, Beijing Tiantan Hospital, Capital Medical University, Beijing, China; 3 Department of Epidemiology and Health Statistics, School of Public Health, Capital Medical University, Beijing, China; 4 Beijing Municipal Key Laboratory of Clinical Epidemiology, Beijing, China; 5 Department of Biostatistics in Gillings School of Global Public Health, The University of North Carolina at Chapel Hill, North Carolina, USA

**Keywords:** Bilirubin, Prognosis, Poor functional outcomes, Acute ischemic stroke, Transient ischemic attack

## Abstract

**Background:**

The prognostic value of serum bilirubin in stroke is controversial, since bilirubin has both neuroprotective and neurotoxic properties. We aimed to investigate the association between serum bilirubin, including total bilirubin (TBIL), direct bilirubin (DBIL) and indirect bilirubin (IBIL) and poor functional outcomes in patients with acute ischemic stroke (AIS) or transient ischemic attack (TIA).

**Methods:**

All patients with AIS or TIA were recruited from the Third China National Stroke Registry. The poor functional outcomes included modified Rankin Scale (mRS) score 2–6 and 3–6 at 3 months and 1 year. Multivariable logistic regression was used to investigate the associations of TBIL, DBIL, and IBIL with poor functional outcomes.

**Results:**

Among 11,121 enrolled patients, the median (interquartile range) of TBIL, DBIL, and IBIL was 13.30 (9.90–17.70), 3.80 (2.70–5.30), and 9.30 (6.70–12.80) µmol/L. After adjustment for conventional confounding factors, patients in the highest TBIL quartile had the highest proportion of mRS score 2–6 at 3 months (odds ratio [OR], 1.37; 95 % confidence interval [CI], 1.19–1.59) and 1 year (OR, 1.31; 95 % CI, 1.13–1.52), and mRS score 3–6 at 3 months (OR, 1.33; 95 % CI, 1.11–1.59) and 1 year (OR, 1.28; 95 % CI, 1.07–1.53), when compared to patients in the lowest TBIL quartile. Similar results were observed for DBIL and IBIL. We also found J-shaped associations between serum bilirubin levels and each outcome.

**Conclusions:**

Elevated levels of serum bilirubin were significantly associated with poor functional outcomes in patients with AIS or TIA at 3 months and 1 year.

**Supplementary Information:**

The online version contains supplementary material available at 10.1186/s12883-021-02398-z.

## Background

Serum bilirubin is the end product of heme metabolism, it can exert both neuroprotective and neurotoxic effect on stroke through mechanisms involved in the development, thus may have an influence on the prognosis of ischemic stroke [1]. Nevertheless, conclusions deprived from previous studies on the association between bilirubin and stroke prognosis were conflicting [2–9]. Some supported a positive association between serum bilirubin and poor functional outcomes, suggesting that serum bilirubin may be a highly toxic substance when high concentrations accumulated within biological tissues [2–5]. While other data supported a negative or a null significant association, suggesting that bilirubin harbors powerful antioxidant properties, higher levels of serum bilirubin might offer a therapeutic advantage in oxidative stress-mediated diseases, such as ischemic stroke [6–10]. This appear paradox between the strong basic evidence and the failure in translation to humans brings great confusion. Possible reasons for the discrepancy may be the small sample size in previous studies and the absence of long-term follow up.

We therefore conducted the current study to investigate the association of between serum bilirubin, including total bilirubin (TBIL), direct bilirubin (DBIL), and indirect bilirubin (IBIL), with poor functional outcomes in patients with acute ischemic stroke (AIS) or transient ischemic attack (TIA) using data from a large, long-term follow-up, prospective registry.

## Materials and methods

### Study population

Data were derived from the Third China National Stroke Registry (CNSR-III), which is a nationwide prospective registry for patients presented to hospitals with acute ischemic cerebrovascular events between August 2015 and March 2018 in China. Participants were consecutively enrolled if meeting the following criteria: (1) age 18 years or older; (2) diagnosis within 7 days of the index event of ischemic stroke or TIA; (3) informed consent from participant or legally authorized representative. The detailed, rationale, and basic description of the CNSR-III have been published previously [11]. A total of 15,166 patients were included from 201 hospitals of 22 provinces. The study protocol was approved by the ethics committee of all participating study centers according to the principles expressed in the Declaration of Helsinki. Written informed consent was provided by all participants or their legal proxies.

### Baseline data collection

Trained research coordinators at each site collected baseline data through a direct interview or medical records, including age, sex, body mass index (BMI, calculated as the weight in kilograms divided by the square of the height in meters), smoking status, medical history, primary diagnosis, the aetiological classification conducted by the Trial of Org 10,172 in Acute Stroke Treatment (TOAST) criteria [12], medication in hospital, and National Institutes of Health Stroke Scale (NIHSS) score.

### Sample collection and measurement of serum bilirubin

Blood samples were collected within 24 h of admission. Plasma specimens were extracted, aliquoted and transported through cold chain to the central laboratory in Beijing Tiantan Hospital and stored at -80℃refrigerator until tests were performed centrally and blindly.

TBIL, DBIL, IBIL, total cholesterol (TC), low-density lipoprotein cholesterol (LDL-C), high-density lipoprotein cholesterol (HDL-C), triglyceride (TG), fasting blood glucose (FBG), serum creatinine, high-sensitivity C-reactive protein (hs-CRP), alanine aminotransferase (ALT), and aspartate aminotransferase (ALT) were tested in the central laboratory of Beijing Tiantan Hospital using Hitachi 7600 automatic biochemistry analyzer (Hitachi, Tokyo, Japan). The estimated glomerular filtration rate (eGFR) was calculated by Chronic Kidney Disease Epidemiology Collaboration equations [13].

### Outcome assessment

Patients were followed up for clinical outcomes at 3 months and 1year after symptom onset. They were interviewed face to face at 3 months and contacted over the telephone by trained research coordinators at 1 year. The poor functional outcomes included modified Rankin Scale (mRS) score 2–6/3–6 at 3 months and 1 year [14–16]. All events were collected by trained research coordinators who were blinded to subjects’ baseline characteristics.

### Statistical analysis

Participants were classified into four groups by TBIL quartiles, DBIL quartiles, and IBIL quartiles, respectively. Continuous variables are presented as median (interquartile range [IQR]) because of the skewed distribution. Categorical variables are presented as frequency and percentage. The non-parametric Wilcoxon or Kruskal-Wallis tests is used to compare group differences for continuous variables and chi-square test is used for categorical variables.

Ordinal logistics regression was applied to estimate the common odds ratio (OR) for a shift in the direct of worse outcome on the mRS score according to serum bilirubin. The association between serum bilirubin and poor functional outcomes were assessed with multivariable binary logistic regression. Variables with *P* < 0.05 and the well-established predictors were selected as confounding variables in the multivariable analyses. The lowest quartile was defined as the reference group. Unadjusted and adjusted ORs and their 95 % confidence intervals (CIs) were calculated. Because 201 hospitals participated in the study, the hospital was treated as clusters in the model and the sandwich estimated were used to account for the correlations. Trends tests were performed using quartiles as ordinal variables. In addition, we used restricted cubic splines to examine the shape of the association between bilirubin and outcomes with five knots (at the 5th, 25th, 50th, 75th, and 95th percentiles) [17]. Reference points for TBIL, DBIL, and IBIL were the median (8.10, 2.05, and 5.10 µmol/L, respectively) of the reference group (the lowest quartile) and OR was adjusted for variables in the multivariable analysis. Additionally, we used C statistics, integrated discrimination improvement (IDI), and net reclassification index (NRI) to evaluate the incremental predictive value of TBIL, DBIL, and IBIL beyond conventional risk factors. To test the robustness of the findings, sensitivity analysis was also performed by excluding patients with cardioembolism or TIA. Considering stroke severity at admission was a strong confounding factor the prognosis of stroke patients, subgroup analysis stratified by stroke severity measured by NIHSS at baseline (NIHSS ≤ 3 for minor ischemic stroke and NIHSS > 3 for moderated to several ischemic stroke) was performed [18], interactions between subgroups were tested using likelihood ratio tests.

Overall, a two-sided *P* < 0.05 was considered statistically significant. All analyses were performed with SAS software version 9.4 (SAS Institute Inc., Cary, NC, USA).

## Results

### Baseline characteristics

Of 15,166 patients, 3,689 participants without TBIL, DBIL, and IBIL and 356 patients without available mRS score at 3 months or 1 year were excluded. Thus, a total of 11,121 patients were included in the final analysis. The baseline characteristics of included and excluded patients were well balanced, except that the included patients had a lower BMI, a higher prevalence of stroke or TIA, peripheral vascular disease and qualifying TIA, a higher proportion of use of cholesterol-lowering agents, antihypertensive agents, and a higher NIHSS, TC, LDL-C, and AST level (Table S1).

Among the 11,121 patients included, the median age was 63.00 years and 7,569 (68.06 %) patients were men. The median level of TBIL, DBIL, and IBIL was 13.30 (IQR, 9.90–17.70), 3.80 (IQR, 2.70–5.30), and 9.30 (IQR, 6.70–12.80) µmol/L. Baseline characteristics according to quartiles of TBIL is presented in Table 1. Compared with patients with lower TBIL, those with higher TBIL were more likely to be men, current smokers, had a higher prevalence of diabetes mellitus, atrial fibrillation/flutter, ischemic stroke, a lower proportion of use of hypoglycemic agents, antiplatelet agents, a higher proportion of use of anticoagulant agents, a higher NIHSS, TC, TG, hs-CRP, ALT, AST level and a lower level of HDL-C.

**Table 1:** Baseline characteristics of included patients according to the level of serum total bilirubin

Variable	Overall	Quartile of total bilirubin, μmol/L				*P* value
		Q1(<9.90)	Q2 (9.90-13.28)	Q3 (13.28-17.67)	Q4 (≥17.67)	
n	11121	2797	2746	2791	2787	
Age, y	63.00(54.00-70.00)	62.00(54.00-70.00)	63.00(54.00-70.00)	63.00(54.00-70.00)	63.00(55.00-71.00)	0.1924
Men, n (%)	7569 (68.06)	1676 (59.92)	1793 (65.29)	1967 (70.48)	2133 (76.53)	<0.0001
BMI, kg/m^2^	24.47(22.49-26.42)	24.34(22.49-26.35)	24.44(22.58-26.37)	24.49(22.76-26.49)	24.49(22.49-26.45)	0.1316
Current smoker	3488 (31.36)	869 (31.07)	862 (31.39)	873 (31.28)	884 (31.72)	0.0001
Medical History, n (%)						
Hypertension	6958 (62.57)	1765 (63.10)	1717 (62.53)	1727 (61.88)	1749 (62.76)	0.8123
Diabetes mellitus	2572 (23.13)	751 (26.85)	644 (23.45)	640 (22.93)	537 (19.27)	<0.0001
Dyslipidemia	858 (7.72)	218 (7.79)	227 (8.27)	219 (7.85)	194 (6.96)	0.3218
Stroke or TIA	2525 (22.70)	681 (24.35)	624 (22.72)	606 (21.71)	614 (22.03)	0.0863
Atrial fibrillation/flutter	743 (6.68)	97 (3.47)	132 (4.81)	181 (6.49)	333 (11.95)	<0.0001
Peripheral vascular disease	76 (0.68)	25 (0.89)	18 (0.66)	17 (0.61)	16 (0.57)	0.4621
Heart failure	68 (4.39)	12 (3.46)	11 (3.09)	19 (4.91)	26 (5.68)	0.2402
Stroke type/Subtype, n (%)						
Ischemic stroke	10339 (92.97)	2554 (91.31)	2530 (92.13)	2617 (93.77)	2638 (94.65)	<0.0001
TIA	782 (7.03)	243 (8.69)	216 (7.87)	174 (6.23)	149 (5.35)	
TOAST, n (%)						
Large-artery atherosclerosis	2811 (25.28)	717 (25.63)	674 (24.54)	718 (25.73)	702 (25.19)	<0.0001
Cardioembolism	662 (5.95)	113 (4.04)	130 (4.73)	150 (5.37)	269 (9.65)	
Small-vessel occlusion	2319 (20.85)	578 (20.66)	590 (21.49)	627 (22.47)	524 (18.80)	
Other determined etiology	149 (1.34)	56 (2.00)	26 (0.95)	27 (0.97)	40 (1.44)	
Undetermined etiology	5180 (46.58)	1333 (47.66)	1326 (48.29)	1269 (45.47)	1252 (44.92)	
Medication in hospital, n (%)						
Cholesterol-lowering agents	10613 (96.12)	2656 (95.51)	2629 (96.51)	2650 (95.77)	2678 (96.71)	0.0584
Antihypertensive agents	5052 (45.76)	1253 (45.06)	1223 (44.90)	1277 (46.15)	1299 (46.91)	0.3879
Hypoglycemic agents	2751 (24.92)	747 (26.86)	688 (25.26)	696 (25.15)	620 (22.39)	0.0016
Antiplatelet agents	10723 (97.12)	2730 (98.17)	2662 (97.72)	2679 (96.82)	2652 (95.77)	<0.0001
Anticoagulant agents	1097 (9.94)	262 (9.42)	229 (8.41)	247 (8.93)	359 (12.96)	<0.0001
NIHSS score on admission	3(1-6)	3(1-5)	3(1-5)	3(1-6)	4(2-7)	<0.0001
Laboratory tests						
TC, mmol/L	4.00(3.33-4.74)	3.94(3.26-4.71)	3.99(3.32-4.72)	4.01(3.35-4.77)	4.03(3.37-4.77)	0.0412
LDL, mmol/L	2.34(1.74-3.00)	2.33(1.75-2.97)	2.31(1.73-3.01)	2.35(1.76-2.99)	2.36(1.73-3.04)	0.5899
HDL, mmol/L	0.93(0.77-1.12)	0.94(0.78-1.15)	0.94(0.79-1.11)	0.93(0.77-1.11)	0.91(0.76-1.10)	0.0028
TG, mmol/L	1.36(1.03-1.87)	1.29(0.99-1.74)	1.33(1.02-1.82)	1.42(1.06-1.95)	1.44(1.09-2.02)	<0.0001
FBG, mmol/L	5.51(4.89-6.88)	5.51(4.89-6.95)	5.50(4.89-6.81)	5.53(4.90-6.95)	5.51(4.88-6.82)	0.5578
eGFR, mL/min/1.73 m^2^	92.99(81.39-101.75)	93.72(79.48-102.24)	92.62(80.80-101.14)	93.06(82.93-101.74)	92.73(82-101.91)	0.2971
hs-CRP, mg/L	1.76(0.80-4.71)	1.75(0.86-4.63)	1.64(0.77-4.25)	1.72(0.79-4.53)	1.90(0.82-5.72)	0.0008
ALT, U/L	18.00(13.00-25.80)	17.00(12.00-24.00)	18.00(13.00-25.00)	18.00(13.00-26.00)	19.00(14.00-27.00)	<0.0001
AST, U/L	19.00(16.00-24.00)	18.05(15.00-23.00)	19.00(15.30-24.00)	19.40(16.00-24.00)	20.10(17.00-26.00)	<0.0001

Continuous variables are expressed as median with interquartile range. Categorical variables are expressed as frequency with percentage

Abbreviations: *ALT* alanine aminotransferase, *AST* aspartate aminotransferase, *BMI* body mass index, *eGFR* estimated glomerular filtration rate, *FBG* fasting blood glucose, *HDL* high-density lipoprotein cholesterol, *hs-CRP* high sensitivity C-reactive protein, *LDL* low-density lipoprotein cholesterol, *NIHSS* The National Institutes of Health Stroke Scale, *TC* total cholesterol, *TG* triglycerides, *TIA* transient Ischemic Attack

### Serum bilirubin and functional outcomes

**Fig. 1:**
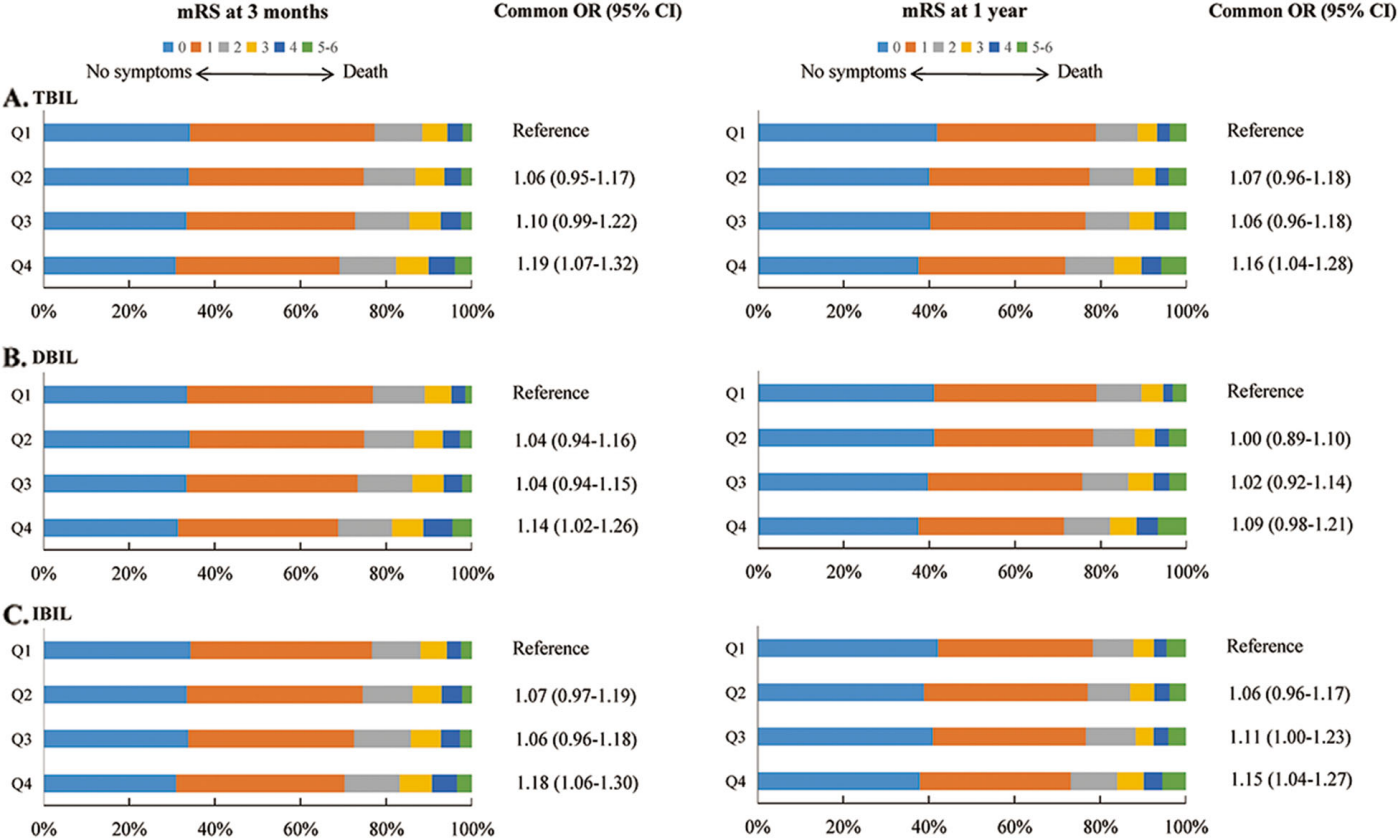
The distribution of mRS scores stratified by (**A**) TBIL, **B** DBIL, and **C** IBIL levels. mRS score ranges from 0 to 6, with 0 indicating no symptoms, 1 no clinically disability, 2 slight disability, 3 moderate disability, 4 moderately severe disability, 5 severe disability and 6 death. In the multivariable ordinal logistic regression, we adjusted for age, sex, history of diabetes, atrial fibrillation/flutter, smoking status, stroke subtype, hypoglycemic agents, antiplatelet agents, baseline National Institutes of Health Stroke Scale score, total cholesterol, high density lipoprotein cholesterol, triglyceride, high sensitivity C-reactive protein, alanine aminotransferase and aspartate aminotransferase

Poor functional outcome (mRS score 2–6) occurred in 2,945 (26.48 %) patients and mRS score 3–6 occurred in 1,581 (14.22 %) at 3 months. A mRS score 2–6 at 1 year assessment occurred in 2,657 patients (23.89 %) and a mRS score 3–6 occurred in 1,498 patients (12.47 %) (Table 2). There was significant shifts in the distribution of mRS score according to the levels of TBIL, DBIL, and IBIL (Fig. 1).

**Table 2:** Adjusted odds ratio (95% confidence interval) for poor functional outcomes according to serum bilirubin levels

Bilirubin	Outcomes	Quartiles	Outcomes at 3 months			Outcomes at 1 year		
			Events, n(%)	Unadjusted	Adjusted^a^	Events, n (%)	Unadjusted	Adjusted^a^
TBIL	mRS score 2-6	Quartile 1	634 (22.67)	Reference	Reference	591 (21.13)	Reference	Reference
		Quartile 2	692 (25.20)	1.19(1.05-1.36)	1.14(0.99-1.32)	620 (22.58)	1.14(0.99-1.30)	1.09(0.95-1.26)
		Quartile 3	759 (27.19)	1.34(1.18-1.53)	1.31(1.14-1.52)	658 (23.58)	1.21(1.06-1.39)	1.17(1.01-1.35)
		Quartile 4	860 (30.86)	1.53(1.35-1.74)	1.37(1.19-1.59)	788 (28.27)	1.51(1.32-1.72)	1.31(1.13-1.52)
		*P* for trend	<0.0001	<0.0001	<0.0001	<0.0001	<0.0001	<0.0001
	mRS score 3-6	Quartile 1	323 (11.55)	Reference	Reference	319 (11.41)	Reference	Reference
		Quartile 2	361 (13.15)	1.19(1.01-1.41)	1.13(0.95-1.36)	338 (12.31)	1.13(0.95-1.34)	1.04(0.87-1.25)
		Quartile 3	405 (14.51)	1.35(1.14-1.59)	1.29(1.08-1.54)	370 (13.26)	1.27(1.08-1.50)	1.18(0.98-1.42)
		Quartile 4	492 (17.65)	1.58(1.35-1.86)	1.33(1.11-1.59)	471 (16.90)	1.59(1.35-1.87)	1.28(1.07-1.53)
		*P* for trend	<0.0001	<0.0001	<0.0001	<0.0001	<0.0001	<0.0001
DBIL	mRS score 2-6	Quartile 1	625 (23.05)	Reference	Reference	569 (20.98)	Reference	Reference
		Quartile 2	701 (25.09)	1.15(1.01-1.32)	1.13(0.97-1.31)	607 (21.73)	1.10(0.96-1.26)	1.05(0.90-1.22)
		Quartile 3	766 (26.62)	1.26(1.10-1.45)	1.18(1.01-1.38)	699 (24.30)	1.33(1.15-1.53)	1.21(1.04-1.42)
		Quartile 4	853 (31.15)	1.61(1.40-1.86)	1.38(1.17-1.62)	782 (28.56)	1.69(1.47-1.95)	1.37(1.17-1.61)
		P for trend	<0.0001	<0.0001	<0.0001	<0.0001	<0.0001	<0.0001
	mRS score 3-6	Quartile 1	297 (10.95)	Reference	Reference	284 (10.47)	Reference	Reference
		Quartile 2	376 (13.46)	1.33(1.12-1.59)	1.29(1.07-1.56)	336 (12.03)	1.23(1.03-1.47)	1.12(0.92-1.35)
		Quartile 3	399 (13.87)	1.39(1.16-1.66)	1.29(1.06-1.56)	390 (13.56)	1.44(1.20-1.72)	1.23(1.01-1.49)
		Quartile 4	509 (18.59)	1.98(1.66-2.37)	1.59(1.30-1.94)	488 (17.82)	1.97(1.64-2.36)	1.40(1.14-1.71)
		*P* for trend	<0.0001	<0.0001	<0.0001	<0.0001	<0.0001	<0.0001
IBIL	mRS score 2-6	Quartile 1	641 (23.30)	Reference	Reference	597 (21.70)	Reference	Reference
		Quartile 2	709 (25.44)	1.14(1.00-1.30)	1.10(0.95-1.27)	638 (22.89)	1.07(0.94-1.22)	1.03(0.90-1.20)
		Quartile 3	769 (27.46)	1.32(1.15-1.50)	1.26(1.09-1.46)	675 (24.11)	1.19(1.04-1.36)	1.13(0.97-1.31)
		Quartile 4	826 (29.68)	1.42(1.24-1.62)	1.30(1.12-1.51)	747 (26.84)	1.35(1.18-1.55)	1.21(1.04-1.41)
		*P* for trend	<0.0001	<0.0001	<0.0001	<0.0001	<0.0001	<0.0001
	mRS score 3-6	Quartile 1	329 (11.96)	Reference	Reference	337 (12.25)	Reference	Reference
		Quartile 2	385 (13.81)	1.18(1.00-1.39)	1.15(0.96-1.38)	362 (12.99)	1.07(0.91-1.26)	1.03(0.86-1.23)
		Quartile 3	398 (14.21)	1.26(1.07-1.49)	1.21(1.01-1.45)	353 (12.61)	1.08(0.91-1.28)	1.00(0.83-1.20)
		Quartile 4	469 (16.85)	1.48(1.25-1.75)	1.31(1.09-1.57)	446 (16.03)	1.43(1.21-1.69)	1.23(1.02-1.48)
		*P* for trend	<0.0001	<0.0001	<0.0001	0.0002	<0.0001	<0.0001

Abbreviations: *DBIL* direct bilirubin, *IBIL* indirect bilirubin, *mRS* modified Rankin Scale, *TBIL* total bilirubin

^a^Adjusted for age, sex, history of diabetes, atrial fibrillation/flutter, smoking status, stroke subtype, TOAST, hypoglycemic agents, antiplatelet agents, anticoagulant agents, baseline National Institutes of Health Stroke Scale score, total cholesterol, high density lipoprotein cholesterol, triglyceride, high sensitivity C-reactive protein, alanine aminotransferase and aspartate aminotransferase

**Fig. 2:**
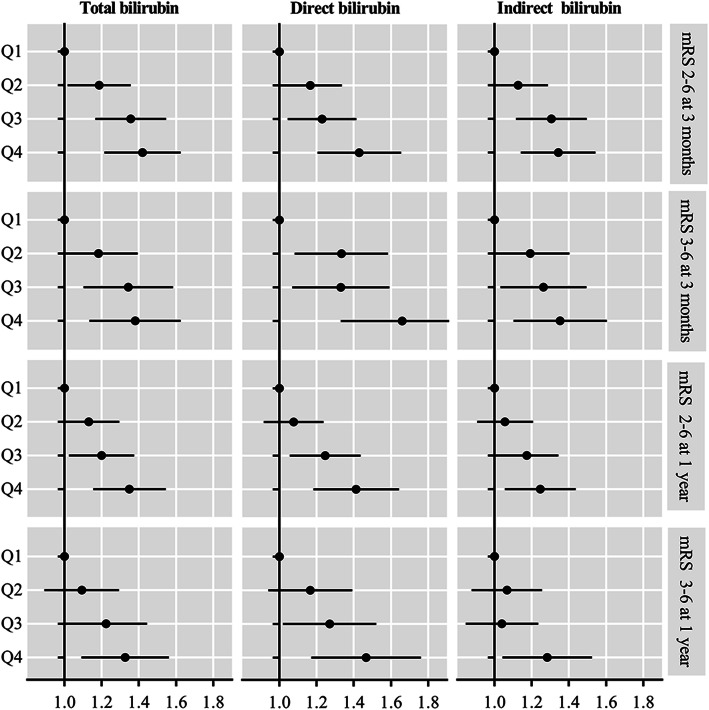
Association between serum bilirubin levels and poor functional outcomes. Adjusted for age, sex, history of diabetes, atrial fibrillation/flutter, smoking status, stroke subtype, hypoglycemic agents, antiplatelet agents, baseline National Institutes of Health Stroke Scale score, total cholesterol, high density lipoprotein cholesterol, triglyceride, high sensitivity C-reactive protein, alanine aminotransferase and aspartate aminotransferase

The associations of TBIL, DBIL, and IBIL with poor functional outcomes are presented in Table 2; Fig. 2. After adjustment for age, sex, history of diabetes, atrial fibrillation/flutter, smoking status, stroke subtype, TOAST, hypoglycemic agents, antiplatelet agents, anticoagulant agents, baseline NIHSS, TC, HDL-C, TG, hs-CRP, ALT and AST, higher TBIL, DBIL, and IBIL levels were associated increased risk of poor functional outcome at 3 months (*P* for trend < 0.0001), the adjusted OR for the quartile 4 subgroup and mRS score 2–6 at 3 months was 1.37 (95 % CI, 1.19–1.59) for TBIL, 1.38 (95 % CI, 1.17–1.62) for DBIL, and 1.30 (95 % CI, 1.12–1.51) for IBIL, respectively. When poor functional was defined as mRS score 3–6, the adjusted OR for the highest vs. the lowest quartile was 1.33 (95 % CI, 1.11–1.59) for TBIL, 1.59 (95 % CI, 1.30–1.94) for DBIL, and 1.31 (95 % CI, 1.09–1.57) for IBIL, respectively. Similar results were observed at 1 year follow-up.

**Fig. 3:**
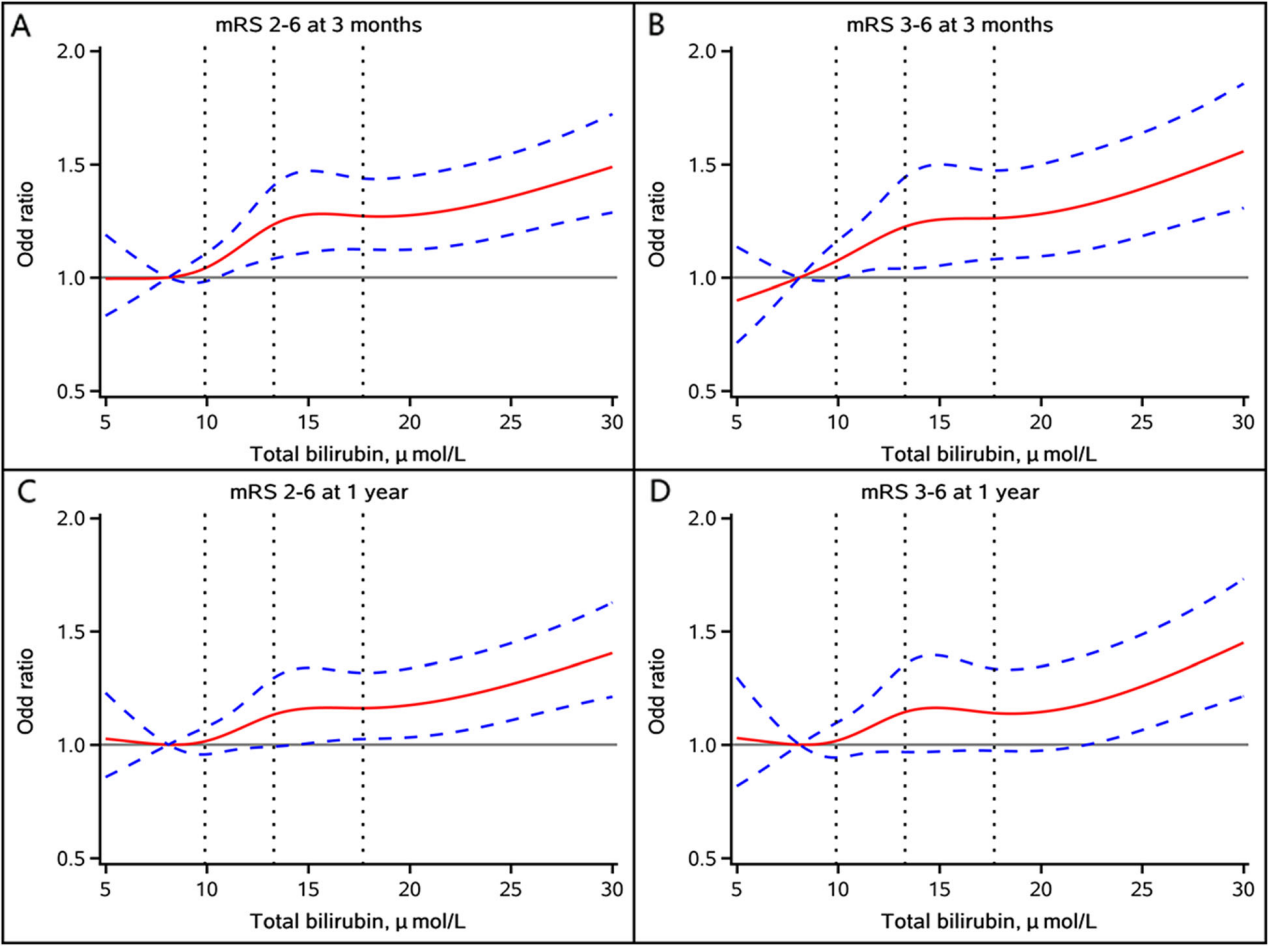
Multivariable-adjusted odds ratio and 95 % confidence interval for (**A**) mRS score 2–6 at 3 months; **B** mRS score 3–6 at 3 months; **C** mRS score 2–6 at 1 year; and **D** mRS score 2–6 at 1 year by total bilirubin level. Red lines represent the adjusted odds ratio and blue dash lines represent 95 % confidence interval. Adjusted for age, sex, history of diabetes, atrial fibrillation/flutter, smoking status, stroke subtype, hypoglycemic agents, antiplatelet agents, baseline National Institutes of Health Stroke Scale score, total cholesterol, high density lipoprotein cholesterol, triglyceride, high sensitivity C-reactive protein, alanine aminotransferase and aspartate aminotransferase

**Fig. 4:**
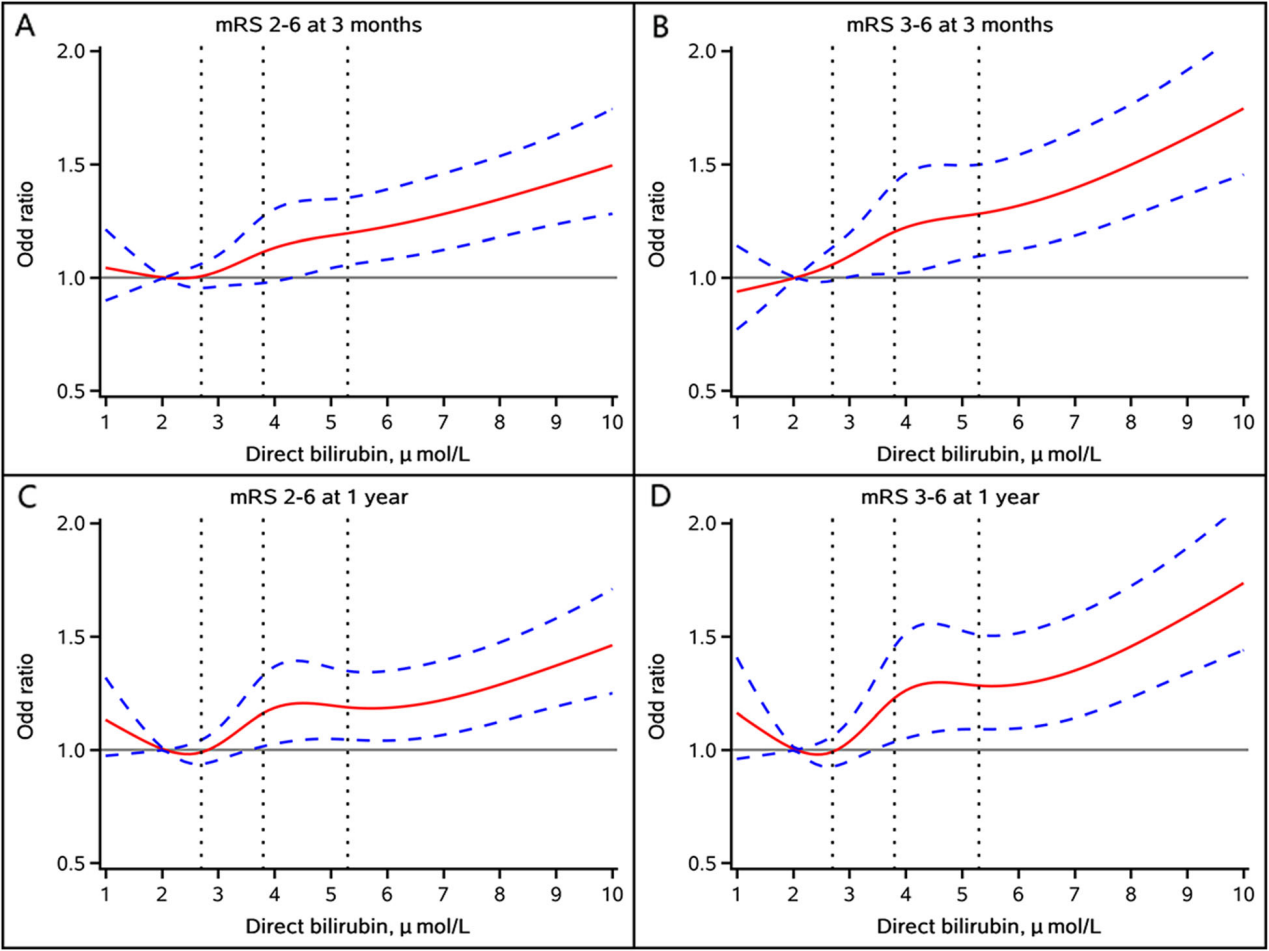
Multivariable-adjusted odds ratio and 95 % confidence interval for (**A**) mRS score 2–6 at 3 months; **B** mRS score 3–6 at 3 months; **C** mRS score 2–6 at 1 year; and **D** mRS score 2–6 at 1 year by direct bilirubin level. Red lines represent the adjusted odds ratio and blue dash lines represent 95 % confidence interval. Adjusted for age, sex, history of diabetes, atrial fibrillation/flutter, smoking status, stroke subtype, hypoglycemic agents, antiplatelet agents, baseline National Institutes of Health Stroke Scale score, total cholesterol, high density lipoprotein cholesterol, triglyceride, high sensitivity C-reactive protein, alanine aminotransferase and aspartate aminotransferase

**Fig. 5:**
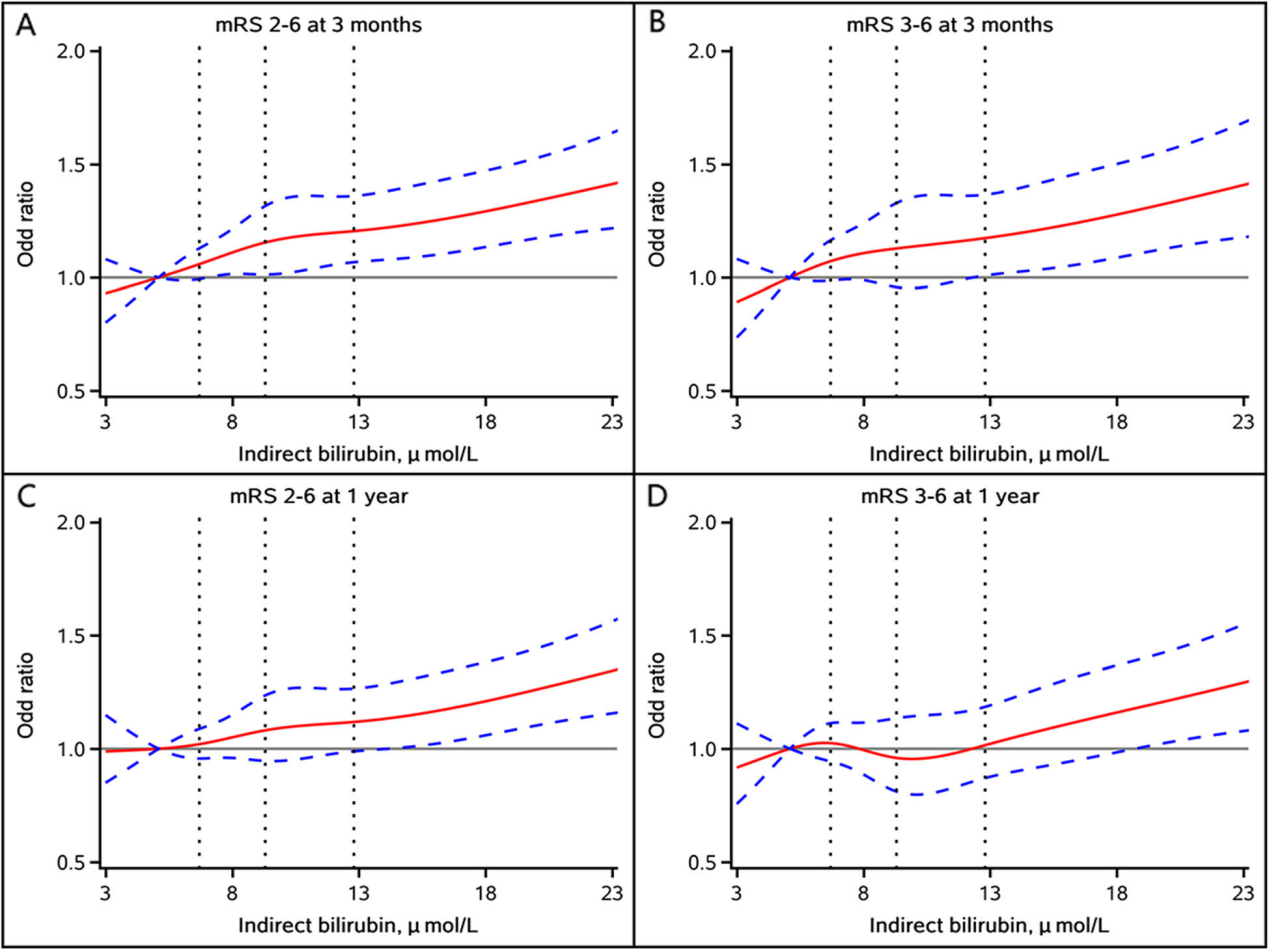
Multivariable-adjusted odds ratio and 95 % confidence interval for (**A**) mRS score 2–6 at 3 months; **B** mRS score 3–6 at 3 months; **C** mRS score 2–6 at 1 year; and (**D**) mRS score 2–6 at 1 year by indirect bilirubin level. Red lines represent the adjusted odds ratio and blue dash lines represent 95 % confidence interval. Adjusted for age, sex, history of diabetes, atrial fibrillation/flutter, smoking status, stroke subtype, hypoglycemic agents, antiplatelet agents, baseline National Institutes of Health Stroke Scale score, total cholesterol, high density lipoprotein cholesterol, triglyceride, high sensitivity C-reactive protein, alanine aminotransferase and aspartate aminotransferase

Multivariable-adjusted spline regression models showed J-shaped associations of TBIL, DBIL, and IBIL levels with poor functional outcomes, including mRS score 2–6 and mRS score 3–6, at 3 months and 1 year follow-up (Figs. 3, 4 and 5).

### Incremental predictive value of bilirubin

We evaluated whether TBIL, DBIL, and IBIL would further increase the predictive value of conventional risk factors (Table 3). For mRS score 2–6 at 3 months as the outcome of interest, the C statistics by the conventional model can significantly improve by addition of TBIL (from 0.762 to 0.763, *P* = 0.0437). Furthermore, the discriminatory power and risk reclassification also appeared to substantially better, the IDI and category-free NRS was 2.07 % (95 % CI, 1.10-3.00 %, *P* < 0.0001) and 8.56 % (95 %CI, 4.33-12.80 %, *P* < 0.0001), respectively. Similar results were found for DBIL, IBIL, mRS score 3–6 as the outcome of interest, and when the time point was set as 1 year.

**Table 3:** Reclassification and discrimination statistics for outcomes within 90 days and 1 year by serum bilirubin

	C statistic		IDI		Category-free NRI	
	Estimate (95% CI)	*P* value	Estimate (95% CI), %	*P* value	Estimate (95% CI), %	*P* value
Outcomes at 3 months						
mRS score 2-6						
Conventional model^a^	0.762(0.752-0.772)		Reference		Reference	
Conventional model+TBIL	0.763(0.753-0.773)	0.0437	2.07(1.10-3.00)	<0.0001	8.56(4.33-12.80)	<0.0001
Conventional model+DBIL	0.762(0.753-0.773)	0.1089	2.12(1.20-3.10)	<0.0001	8.43(4.18-12.68)	0.0001
Conventional model+IBIL	0.763(0.753-0.773)	0.0443	1.52(0.07-2.40)	0.0004	7.17(2.94-11.41)	0.0010
mRS score 3-6						
Conventional model^a^	0.780(0.768-0.792)		Reference		Reference	
Conventional model+TBIL	0.782(0.770-0.794)	0.0252	2.44(1.10-3.70)	0.0003	10.53(5.15-15.90)	0.0001
Conventional model+DBIL	0.782(0.770-0.794)	0.0432	4.50(2.70-6.30)	<0.0001	10.56(5.18-15.95)	0.0001
Conventional model+IBIL	0.781(0.769-0.793)	0.0431	1.18(0.20-2.10)	0.0138	8.03(2.66-13.40)	0.0037
Outcomes at 1 year						
mRS score 2-6						
Conventional model^a^	0.738(0.727-0.749)		Reference		Reference	
Conventional model+TBIL	0.739(0.728-0.750)	0.0497	1.64(0.70-2.50)	0.0003	7.23(2.85-111.62)	0.0014
Conventional model+DBIL	0.739(0.728-0.749)	0.1763	1.84(0.09-2.80)	0.0001	6.10(1.68-10.48)	0.0072
Conventional model+IBIL	0.739(0.728-0.750)	0.0379	1.13(0.40-1.90)	0.0034	5.64(2.26-10.03)	0.0127
mRS score 3-6						
Conventional model^a^	0.776(0.764-0.790)		Reference		Reference	
Conventional model+TBIL	0.778(0.765-0.791)	0.0667	1.82(0.06-3.10)	0.0040	6.16(0.69-11.63)	0.0239
Conventional model+DBIL	0.779(0.766-0.792)	0.0319	3.79(2.00-5.60)	<0.0001	8.71(3.21-14.21)	0.0021
Conventional model+IBIL	0.777(0.764-0.790)	0.1432	0.76(0.01-1.60)	0.0378	4.77(0.70-10.24)	0.0415

Abbreviations: *CI* confidence interval, *DBIL* direct bilirubin, *IBIL* indirect bilirubin, *IDI* integrated discrimination improvement, *mRS* modified Rankin Scale, *NRI* net reclassification index, *TBIL* total bilirubin

^a^Adjusted for age, sex, history of diabetes, atrial fibrillation/flutter, smoking status, stroke subtype, hypoglycemic agents, antiplatelet agents, baseline National Institutes of Health Stroke Scale score, total cholesterol, high density lipoprotein cholesterol, triglyceride, high sensitivity C-reactive protein, alanine aminotransferase and aspartate aminotransferase

### Sensitivity and subgroup analysis

Sensitivity analysis by excluding patients with cardioembolism or TIA showed consistent results with the main analysis (Table S2). Subgroup analysis for the association of serum bilirubin and poor functional outcomes stratified by stroke severity is shown in Table S2. The adjusted OR for the quartile 4 subgroup and mSR score 2–6 at 3 months was 1.13 (95 % CI, 0.88–1.44) for minor ischemic stroke, and 1.52 (95 % CI, 1.27–1.84) for moderate to severe ischemic stroke. However, there was no significant interaction between stroke severity and TBIL for the risk of mRS score 2–6 (*P* for interaction = 0.0938), indicating the association between TBIL and poor functional outcome was consistent across different stroke severity subgroups. The same results were observed for mRS score 3–6, when the time point was set as 1 year, and when DBIL or IBIL was the exposure.

## Discussion

The main finding of this study was that higher levels of serum bilirubin, including TBIL, DBIL, and IBIL were all independently associated with poor functional outcomes in patients with AIS or TIA at 3 months and 1 year. These associations did not modified by stroke severity.

Evidence on the prognostic value of serum bilirubin in ischemic stroke is still controversial. The Acute Inflammatory Stroke Study with 142 stroke patients and the a cross-sectional prospective descriptive study with 275 ischemic stroke patients showed serum bilirubin level was significantly associated with mortality in the acute phase of ischemic stroke patients [4, 5]. Study of Kurzepa et al. with 43 patients reported that level of TBIL in the acute phase of ischemic stroke proved to be a bad prognostic factor for both early neurological status and for long term neurological functions measured at 3 months after stroke onset [2]. Similarly, a cross-sectional study conducted in Peshawar revealed that higher level of bilirubin was associated with increased poor functional outcomes measured by mRS score in patients with ischemic stroke [19]. This finding was also observed in the analysis on 73 cases of large-artery atherosclerotic stroke, which indicated that the level of TBIL, DBIL, and IBIL was positively associated with poor functional outcomes on day 30 of hospitalization and discharge [3]. While not all previous studies supported the positive association between serum bilirubin and stroke prognosis. A cross-sectional study using data from the National Health and Nutrition Examination Survey showed per 1.71µmol/L (0.1 mg/dL) increment in TBIL was associated with a 10 % reduced odds of an adverse stroke outcome among participants with a history of stroke. Not surprisingly, three studies even showed there was no significant association between levels of serum bilirubin and short-term clinical outcomes among patients with AIS [7–9].

Except the dual effect of serum bilirubin, other possible reasons for these inconsistent conclusions may include the research design, small sample size, as well as lack of long-term follow-up. To address these knowledge gaps and methodological limitations, we recruited patients with AIS or TIA from a large, prospective, and long-term study, which can provide more statistical power. Our results showed that higher levels of TBIL, DBIL, and IBIL were significantly associated with poor functional outcomes of stroke at 3 months and 1 year follow-up, the addition of bilirubin to the conventional risk model had an incremental on the predictive value for poor functional outcomes after ischemic stroke. The findings are in concordance with prior investigations regarding bilirubin as predictor of stroke and provide important insights into the relationship between bilirubin and stroke prognosis.

It was proposed serum bilirubin has also been reported to be associated with stroke severity at admission, which was a strong confounding factor influencing the prognosis of stroke patients, patients with severe stroke have a high risk of disabilities [20–22]. While our subgroup analysis stratified by stroke severity showed that there was no significant interaction between stroke severity and bilirubin in relation to the risk of poor functional outcomes of stroke, indicating the relationship between bilirubin and poor functional outcomes were consistent across difference stroke severity subgroups, and that patients with minor and moderate to severe ischemic stroke had the same risk of poor functional outcomes with elevated bilirubin levels.

The plausible mechanisms responsible for the association between serum bilirubin and poor functional outcomes of stroke are summarized as follows. Bilirubin has both neuroprotective and neurotoxic capacities [1]. When bilirubin levels are in the normal range, it may an important endogenous anti-inflammatory and antioxidant molecule [23, 24]. Contrarily, pathological levels of bilirubin serve as an indicator of severe brain injury, a high concentration of unconjugated bilirubin can be cytotoxic, affecting the permeability of the mitochondrial membrane as well as damaging mitochondrial function and the activity of astrocytes, thereby resulting in neurocyte apoptosis [25–27]. After stroke occurs, the negative influence of high levels of serum bilirubin on patients’ outcome possibly reflects the intensity of initial oxidative stress. Patients with AIS or TIA with higher levels of bilirubin had larger cerebral infarcts, more prominent brain oedema and more severe reperfusion injuries with poorer functional outcomes than those with lower bilirubin levels [3, 28].

The strengths of our study include that the study is a multicenter prospective registry with a large sample size, which resulted in sufficient statistical power. However, our study also had some limitations. First, this study only monitored the baseline bilirubin levels and did not examine the dynamic changes in bilirubin, which may have provided more valuable information regarding understand the mechanism underlying the associations. Second, we excluded patients with missing data on serum bilirubin or mRS, which may lead to selection bias. Third, we did not record detailed information about the treatment on patients with impaired liver function. Specific treatment on patients with high bilirubin levels needed further investigations to explore. Finally, all patients were Chinese, this, our findings may not be generalizable to other races and ethnicities. Finally, some unmeasured or residual confounding effects may still exist because of the nature of the observational study.

## Conclusions

In conclusion, higher levels of bilirubin (TBIL, DBIL, and IBIL) were associated with poor functional outcomes in patients with AIS or TIA at 3 months and 1 year. This findings may promote the potential use of bilirubin in clinical practice as markers for stroke outcomes.

## Supplementary Information

**Additional file 1: Table S1. **Baseline characteristics of included and excluded patients. **Table S2.** Association between serum bilirubin and poor functional outcomes after excluding patients with cardioembolism or TIA. **Table S3.** Association between high serum bilirubin (quartile 4) and poor function outcomes stratified by stroke severity measured by NIHSS.

## Data Availability

Data are available to researchers on request for purposes of reproducing the results or replicating the procedure by directly contacting the corresponding author.
