# Supplementary material for: Serum bilirubin levels are associated with poor functional outcomes in patients with acute ischemic stroke or transient ischemic attack
Source: BMC Neurol. 2021 Sep 27;21:373. doi: 10.1186/s12883-021-02398-z (PMC8493700; doi:10.1186/s12883-021-02398-z)
Supplement: Supplementary file 1 — Additional file 1: Table S1. Baseline characteristics of included and excluded patients. Table S2. Association between serum bilirubin and poor functional outcomes after excluding patients with cardioembolism or TIA. Table S3. Association between high serum bilirubin (quartile 4) and poor function outcomes stratified by stroke severity measured by NIHSS. [file 12883_2021_2398_MOESM1_ESM.docx]

**Serum bilirubin levels are associated with poor functional outcomes in patients with acute ischemic stroke or transient ischemic attack**

**Authors**

Quping Ouyang, PhD^1,2*^; Anxin Wang, PhD^1,2*^; Xue Tian, MS^3,4^; Yingting Zuo, PhD^3,4^; Zhimeng Liu, BS^5^; Qin Xu, PhD^1,2^; Xia Meng, MD, PhD^1,2^; Pan Chen, PhD^1,2^; Hao Li, MD, PhD^1,2^; Yongjun Wang, MD^1,2^

* Contributed equally to this work

**Affiliations**

^1^ China National Clinical Research Center for Neurological Diseases, Beijing Tiantan Hospital, Capital Medical University, Beijing, China;

^2^ Department of Neurology, Beijing Tiantan Hospital, Capital Medical University, Beijing, China;

^3^ Department of Epidemiology and Health Statistics, School of Public Health, Capital Medical University, Beijing, China;

^4^ Beijing Municipal Key Laboratory of Clinical Epidemiology, Beijing, China;

^5^ Department of Biostatistics in Gillings School of Global Public Health, The University of North Carolina at Chapel Hill, North Carolina, USA.

**^#^Corresponding author**

**Yongjun Wang, MD**

China National Clinical Research Center for Neurological Diseases, Beijing Tiantan Hospital, Capital Medical University, No.119 South 4th Ring West Road, Fengtai District, Beijing, 100070, China

Tel: 0086-010-59978538

Fax: 0086-010-59978538

Email: [yongjunwang@ncrcnd.org.cn](mailto:yongjunwang@ncrcnd.org.cn)

**Table S1. Baseline characteristics of included and excluded patients**

| Characteristics | Excluded | Included | *P* value |
| --- | --- | --- | --- |
| n | 4045 | 11121 |  |
| Age, y | 62.00(54.00-70.00) | 63.00(54.00-70.00) | 0.2293 |
| Men, n(%) | 2795 (69.10) | 7569 (68.06) | 0.2246 |
| BMI, kg/m^2^ | 24.62(22.86-26.73) | 24.47(22.49-26.42) | <0.0001 |
| Medical History |  |  |  |
| Hypertension | 2536 (62.69) | 6958 (62.57) | 0.8851 |
| Diabetes mellitus | 938 (23.19) | 2572 (23.13) | 0.9365 |
| Dyslipidemia | 333 (8.23) | 858 (7.72) | 0.2950 |
| Stroke or TIA | 830 (20.52) | 2525 (22.70) | 0.0041 |
| Atrial fibrillation/flutter | 276 (6.82) | 743 (6.68) | 0.7571 |
| Peripheral vascular disease | 42 (1.04) | 76 (0.68) | 0.0278 |
| Heart failure | 26 (4.49) | 68 (4.39) | 0.9222 |
| Stroke type/Subtype |  |  |  |
| Ischemic stroke | 3807 (94.12) | 10339 (92.97) | 0.0126 |
| TIA | 238 (5.88) | 782 (7.03) |  |
| TOAST |  |  |  |
| Large-artery atherosclerosis | 1045 (25.83) | 2811 (25.28) | 0.0958 |
| Cardioembolism | 255 (6.30) | 662 (5.95) |  |
| Small-vessel occlusion | 846 (20.91) | 2319 (20.85) |  |
| Other determined etiology | 33 (0.82) | 149 (1.34) |  |
| Undetermined etiology | 1866 (46.13) | 5180 (46.58) |  |
| Current smoker | 1264 (31.25) | 3488 (31.36) | 0.0990 |
| Medication in hospital |  |  |  |
| Cholesterol-lowering agents | 3893 (97.03) | 10613 (96.12) | 0.0083 |
| Antihypertensive agents | 1948 (48.55) | 5052 (45.76) | 0.0023 |
| Hypoglycemic agents | 1041 (25.95) | 2751 (24.92) | 0.1977 |
| Antiplatelet agents | 3890 (96.96) | 10723 (97.12) | 0.6048 |
| Anticoagulant agents | 449 (11.19) | 1097 (9.94) | 0.0248 |
| NIHSS score on admission | 3(1-6) | 3(1-6) | 0.0112 |
| Lipid level |  |  |  |
| TC, mmol/L | 3.90(3.28-4.66) | 4.00(3.33-4.74) | 0.0013 |
| LDL, mmol/L | 2.24(1.69-2.90) | 2.34(1.74-3.00) | <0.0001 |
| HDL, mmol/L | 0.94(0.78-1.12) | 0.93(0.77-1.12) | 0.5182 |
| TG, mmol/L | 1.37(1.04-1.87) | 1.36(1.03-1.87) | 0.6062 |
| FBG, mmol/L | 5.59(4.90-6.90) | 5.51(4.89-6.88) | 0.1715 |
| eGFR, mL/min/1.73 m^2^ | 93.45(81.95-102.48) | 92.99(81.39-101.75) | 0.0912 |
| hs-CRP, mg/L | 1.84(0.87-4.75) | 1.76(0.80-4.71) | 0.1232 |
| ALT, U/L | 18.00(13.00-26.00) | 18.00(13.00-25.80) | 0.4816 |
| AST, U/L | 19.00(15.20-24.00) | 19.00(16.00-24.00) | 0.0073 |

Abbreviations: ALT=alanine aminotransferase; AST=aspartate aminotransferase; BMI=body mass index; eGFR=estimated glomerular filtration rate; FBG=fasting blood glucose; HDL=high-density lipoprotein cholesterol; hs-CRP=high sensitivity C-reactive protein; LDL=low-density lipoprotein cholesterol; NIHSS=The National Institutes of Health Stroke Scale; TC=total cholesterol; TG=triglycerides; TIA=transient Ischemic Attack. Continuous variables are expressed as median with interquartile range. Categorical variables are expressed as frequency with percentage.

Table S2. Association between serum bilirubin and poor functional outcomes after excluding patients with cardioembolism or TIA

| Outcomes | Index | Q1 | Q2 | Q3 | Q4 | *P* for trend |
| --- | --- | --- | --- | --- | --- | --- |
| mRS 2-6 at 3 months | TBIL | Reference | 1.14(0.94-1.38) | 1.30(1.08-1.57) | 1.34(1.11-1.62) | <0.0001 |
|  | DBIL | Reference | 1.11(0.95-1.30) | 1.18(1.00-1.38) | 1.40(1.18-1.66) | <0.0001 |
|  | IBIL | Reference | 1.14(0.98-1.32) | 1.28(1.10-1.49) | 1.33(1.13-1.56) | <0.0001 |
| mRS 3-6 at 3 months | TBIL | Reference | 1.15(0.99-1.33) | 1.32(1.14-1.54) | 1.40(1.20-1.63) | <0.0001 |
|  | DBIL | Reference | 1.22(1.00-1.48) | 1.25(1.02-1.53) | 1.59(1.29-1.97) | <0.0001 |
|  | IBIL | Reference | 1.22(1.01-1.47) | 1.24(1.03-1.51) | 1.34(1.11-1.63) | <0.0001 |
| mRS 2-6 at 1 year | TBIL | Reference | 1.11(0.95-1.29) | 1.18(1.01-1.38) | 1.35(1.16-1.58) | <0.0001 |
|  | DBIL | Reference | 1.04(0.89-1.22) | 1.22(1.03-1.43) | 1.42(1.20-1.69) | <.0001 |
|  | IBIL | Reference | 1.06(0.91-1.23) | 1.17(1.00-1.37) | 1.26(1.08-1.48) | <.0001 |
| mRS 3-6 1 year | TBIL | Reference | 1.07(0.88-1.29) | 1.20(0.99-1.45) | 1.31(1.08-1.58) | <0.0001 |
|  | DBIL | Reference | 1.12(0.92-1.38) | 1.21(0.99-1.49) | 1.43(1.15-1.77) | <0.0001 |
|  | IBIL | Reference | 1.05(0.87-1.27) | 1.02(0.84-1.24) | 1.28(1.05-1.55) | <0.0001 |

Abbreviations: DBIL= direct bilirubin; IBIL=indirect bilirubin; mRS= modified Rankin Scale; TBIL= total bilirubin; TIA= transient ischemic attack.

Table S3. Association between high serum bilirubin (quartile 4) and poor function outcomes stratified by stroke severity measured by NIHSS

| Outcomes | Variable | TBIL | |  | DBIL | |  | IBIL | |
| --- | --- | --- | --- | --- | --- | --- | --- | --- | --- |
|  |  | HR (95% CI) | *P*_interaction_ |  | HR (95% CI) | *P*_interaction_ |  | HR (95% CI) | *P*_interaction_ |
| mRS score 2-6 at 3 months | NIHSS≤3 | 1.13(0.88-1.44) | 0.0938 |  | 1.11(0.84-1.45) | 0.0633 |  | 1.09(0.84-1.41) | 0.1167 |
|  | NIHSS>3 | 1.52(1.27-1.84) |  |  | 1.54(1.25-1.90) |  |  | 1.43(1.18-1.73) |  |
| mRS score 3-6 at 3 months | NIHSS≤3 | 1.00(0.70-1.43) | 0.4082 |  | 1.00(0.66-1.48) | 0.0941 |  | 1.03(0.71-1.50) | 0.7274 |
|  | NIHSS>3 | 1.44(1.16-1.78) |  |  | 1.85(1.45-2.36) |  |  | 1.39(1.12-1.73) |  |
| mRS score 2-6 at 1 year | NIHSS≤3 | 1.15(0.91-1.46) | 0.1540 |  | 1.19(0.91-1.55) | 0.0716 |  | 1.02(0.80-1.30) | 0.1505 |
|  | NIHSS>3 | 1.45(1.27-1.84) |  |  | 1.50(1.21-1.86) |  |  | 1.36(1.12-1.66) |  |
| mRS score 3-6 at 1 year | NIHSS≤3 | 1.15(0.83-1.60) | 0.2820 |  | 1.15(0.79-1.68) | 0.3291 |  | 1.05(0.75-1.49) | 0.5725 |
|  | NIHSS>3 | 1.30(1.04-1.63) |  |  | 1.47(1.15-1.90) |  |  | 1.28(1.02-1.61) |  |

Abbreviations: CI, confidence interval; DBIL, direct bilirubin; HR, hazard ratio; IBIL, indirect bilirubin; mRS, modified Rankin Scale; NIHSS, National Institutes of Health Stroke Scale; TBIL, total bilirubin.

* Adjusted for age, sex, history of diabetes, atrial fibrillation/flutter, smoking status, stroke subtype, hypoglycemic agents, antiplatelet agents, total cholesterol, high density lipoprotein cholesterol, triglyceride, high sensitivity C-reactive protein, alanine aminotransferase and aspartate aminotransferase.
